# Genomic analysis of firework fear and noise reactivity in standard poodles

**DOI:** 10.1186/s40575-023-00125-0

**Published:** 2023-03-08

**Authors:** Karin Westereng Handegård, Linn Mari Storengen, Dina Joergensen, Frode Lingaas

**Affiliations:** Department of Preclinical Sciences and Pathology, Faculty of Veterinary Medicine, Norwegian University of Life Sciences, P.O. Box 5003, 1432 Ås, Norway

**Keywords:** Noise reactivity, GWAS, Dog, Fear of fireworks, Behavior, Behavioral genetics, Genome heritability, SNP-based heritability

## Abstract

**Background:**

Fear of firework noises and other loud, sudden noises (noise reactivity) is a significant problem for many dogs and may have a negative effect on both welfare and, in severe cases, the life expectancy of dogs. A wide range of behavior traits, including fear-related behaviors, have high heritability estimates in dogs. The aim of this study was to estimate genomic heritability for fear of fireworks and loud noises in dogs.

**Results:**

A genomic heritability estimate was performed based on genome-wide SNPs from standard poodles with records of fear of fireworks and noise reactivity. The study was based on questionnaires answered by owners, who also volunteered to return a cheek swab from their dog for DNA analyses. SNP-based heritability was estimated to be 0.28 for firework fear and 0.16 for noise reactivity. We also identified an interesting region on chromosome 17 that was weakly associated with both traits.

**Conclusions:**

We have estimated low to medium genomic heritabilities for fear of fireworks and noise reactivity in standard poodles. We have also identified an interesting region on chromosome 17, which harbors genes that have been shown to be involved in different psychiatric traits with anxiety components in humans. The region was associated with both traits; however, the association was weak and need further verification from other studies.

**Supplementary Information:**

The online version contains supplementary material available at 10.1186/s40575-023-00125-0.

## Abstract (summary)

The prevalence of fear of fireworks and loud noises is very high in many dog ​​breeds and can be a serious problem for both the dogs and the owners. A genetic study of fear of fireworks and fear of other loud noises was conducted on standard poodles. The study was based on owner questionnaires, where the dog owners scored their dogs’ degree of fearfulness from 1 (not fearful) to 5 (very fearful). We estimated genomic heritabilities of 28% for fear of fireworks and 15% for noise reactivity. We also identified a region on chromosome 17 with a possible association with the two traits. This genomic region contains genes of interest in human anxiety-related disorders. Genomic studies in dog breeds where the individuals with and without fear-associated phenotypes can be clearly distinguished, based on owner records, may provide opportunities for the implementation of methods for genomic selection for those fear-associated traits. The results of this study may be helpful for standard poodle breeders in their selection of breeding animals, which ultimately will contribute to better animal welfare.

**Supplementary Information:**

The online version contains supplementary material available at 10.1186/s40575-023-00125-0.

## Background

Studies have shown that up to 30% of dogs of some breeds, including the standard poodle, show a strong or extreme fear of loud noises and/or fireworks [1–6]. Fearfulness and anxiety disorders constitute a large proportion of behavioral problems in both family dogs and working dogs, and noise reactivity is a large part of these problems [1–3, 5]. Noise reactivity is a complex trait with a wide spectrum of phenotypes, and is likely affected by both environmental factors, as well as heritage. The etiology of fear and anxiety is poorly understood. While fear is a natural response to potentially dangerous stimuli or situations, and is necessary for survival [7–11], excessive fear responses out of context are problematic and may be pathological [12]. Anxiety and fear can also have a strong negative effect on animal welfare [13], as well as the relationship between the owner and the dog [14, 15]. In severe cases, anxiety and fear may impact the life expectancy of the dog [16, 17].

Studies from human behavior research have estimated heritabilities for many personality and behavior traits to be between 20 and 70% [18–20]. These traits include aggressiveness, and disorders like social anxiety and major depression disorder [21–24]. Identifying specific genes associated with behavior traits has, however, proven to be difficult. Several behavior traits, including a range of fear phenotypes, have been shown to have high heritability also in dogs. For example, Goddard and Beilharz found heritability estimates of 0.46 for fearfulness in guide dogs, and Ruefenacht et al. found heritability estimated to 0.23 for reaction to gunfire, similar results have been reported by other researchers [25–28]. The relatively high heritability of behavior traits in dogs is also supported by the variation in prevalence of different behaviors between breeds [29, 30]. Despite the many studies in both humans and several other species, there is limited information about the genetic architecture of phobias, anxieties, and fearful behavior [12, 31, 32].

In recent years, hundreds of human genome-wide association studies (GWAS) have provided new information about genetic associations to disorders like general anxiety, major depressive disorder, schizophrenia, autism spectrum disorders and bipolar disorder and others, indicating that most behavior traits have a complex genetic background where many different loci may be involved [33–37]. GWAS in dogs has successfully identified candidate markers and genes for several behavior traits [38–41] including fearfulness [42–44]. Studies have found significant differences in the frequency of noise reactivity between dog breeds, which suggest this trait has a relevant genetic component [1, 29, 45].

The evolutionary bottlenecks and breeding for specific traits have caused purebred dogs to have lower genetic diversity with longer linkage disequilibrium (LD) compared to humans [46–48]. The accumulation of risk alleles associated with specific behaviors, as well as the limited genetic heterogeneity within breeds, makes the dog a good model for identifying associated loci for complex traits, even with a limited sample size.

The aim of this study was to estimate the genomic (SNP-based) heritability of fear of fireworks and loud noises in standard poodles and search for potential genomic regions associated with these traits.

## Results

### Heritability estimates

The genomic heritability estimates were 0.28 (SE 0.10) for firework fear and 0.16 (SE 0.10) for noise reactivity, with a high genetic correlation between the two traits, 0.99 (SE 0.05).

### Genome-wide association study

**Fig. 1:**
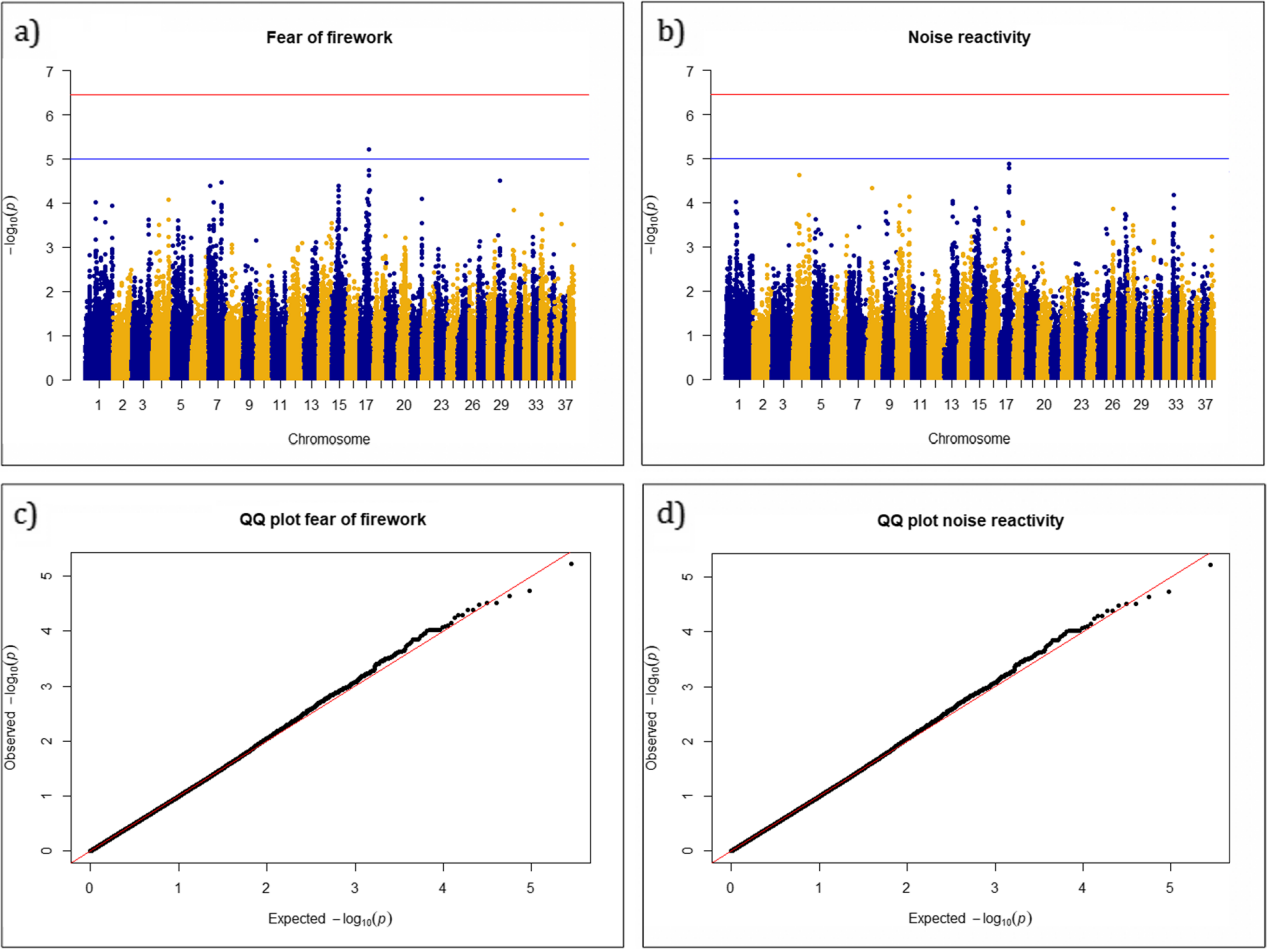
Manhattan plots for **a** firework fear and **b** noise reactivity. A suggestive line (blue) was placed at *p* = 2 × 10^−05^, based on Bonferroni adjustment using the number of independent haplotype blocks (Karlsson et al., 2013). The more conservative GWAS significance line (red) was placed at *p* = 3.54 × 10^− 07^ using the total number of tested SNPs (141.174 SNPs after QC). Quantile-quantile plots for **c** firework fear and **d** noise reactivity. Lambda was calculated at 1.0 for both traits

**Fig. 2:**
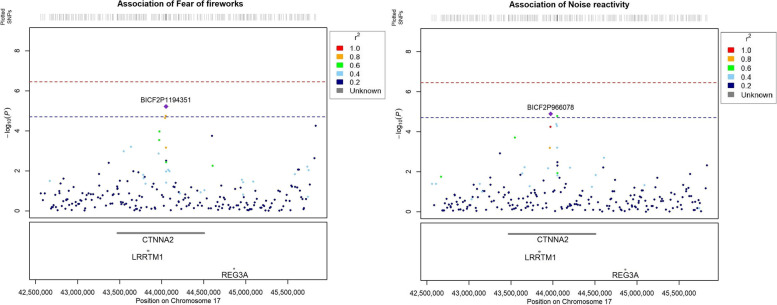
Chromosomal position of top associated SNPs for firework fear and noise reactivity, LD to top SNP is indicated with different colors. Interesting genes in the region are also included. The plots were created using LocusZoom-like [49]

Mixed linear models for the phenotypes firework fear and noise reactivity each identified suggestive association (*p* < 2 × 10^− 05^) to a region on chromosome 17 (CFA17) (Fig. 1). For firework fear the top SNP (BICF2P1194351) was found in position 44.487.783 (*p* = 6.065 × 10^− 06^) and the top SNP for noise reactivity (BICF2P966078) in position 44.409.723 (Table 1). Top SNPs for both traits are located in the same chromosomal region, see Fig. 2. The SNPs are in high level of LD with the neighboring SNPs. Both top SNPs are within the same intron of the Catenin alpha 2 gene (CTNNA2), and in < 1 Mb range of two other genes; Leucine rich repeat transmembrane neuronal 1 (LRRTM1) and Regenerating islet-derived protein III-alpha (REG3A) (Table 2). As shown in Fig. 2, the LD decays around 1.5 Mb on each side of the top SNPs, giving a region/LD block of about 3 Mb (2.973.859). The relevant genes (CTNNA2, LRRTM1, REG3A) within this region are depicted in Fig. 2. CTNNA2 and LRRTM1 are within the highest LD region and would be the most likely candidate genes (Fig. 2, Table 2). SNPs on chromosomes 7 and 15 also reach near-suggestive *p*-values (Fig. 1). All genomic positions are given according to the assembly GSD_1.0 (CanFam4) [50].

**Table 1:** Top SNPs on chromosome 17 with position, *p*-values, risk alleles and allele frequency in the case group and the control group for firework fear and noise reactivity

	SNP	CFA position	*P*-value	Risk allele	Allele frequency		
					Cases	Controls	OR
*Firework fear*	BICF2P1194351	44.487.783	6.065 × 10^−06^	A	0.400	0.246	2.04
	BICF2P602076	44.485.033	1.8264 × 10^−05^	G	0.331	0.201	1.96
	BICF2P350894	44.477.990	2.3265 × 10^−05^	A	0.337	0.206	1.96
	BICF2P1264633	46.831.173	5.1340 × 10^−05^	C	0.226	0.133	1.91
*Noise reactivity*	BICF2P966078	44.409.723	1.2807 × 10^−05^	G	0.300	0.155	2.30
	BICF2P1194351	44.487.783	1.6688 × 10^−05^	A	0.419	0.263	2.02
	BICF2P350894	44.477.990	4.2310 × 10^−05^	A	0.356	0.225	1.90
	BICF2P602076	44.485.033	5.2879 × 10^−05^	G	0.348	0.221	1.88

**Table 2:** Genes in the nearby region of top SNPs. The distance between top SNPs of the two traits is 0.08 Mb

Genes in region	Association summary	Distance from top SNP Fireworks / Noise
*CTNNA2*	Canine: Canine compulsive disorder Human: Excitement seeking, ADHD, Schizophrenia, Bipolar disorder	0.00 Mb / 0.00 Mb
*LRRTM1*	Mice: claustrophobic-like behavior in knock-out-mice Human: Schizophrenia	0.21 Mb / 0.13 Mb
*REG3A*	Human: Gastrointestinal cancers, (no reported association to behavior-traits)	0.81 Mb / 0.89 Mb

**Fig. 3:**
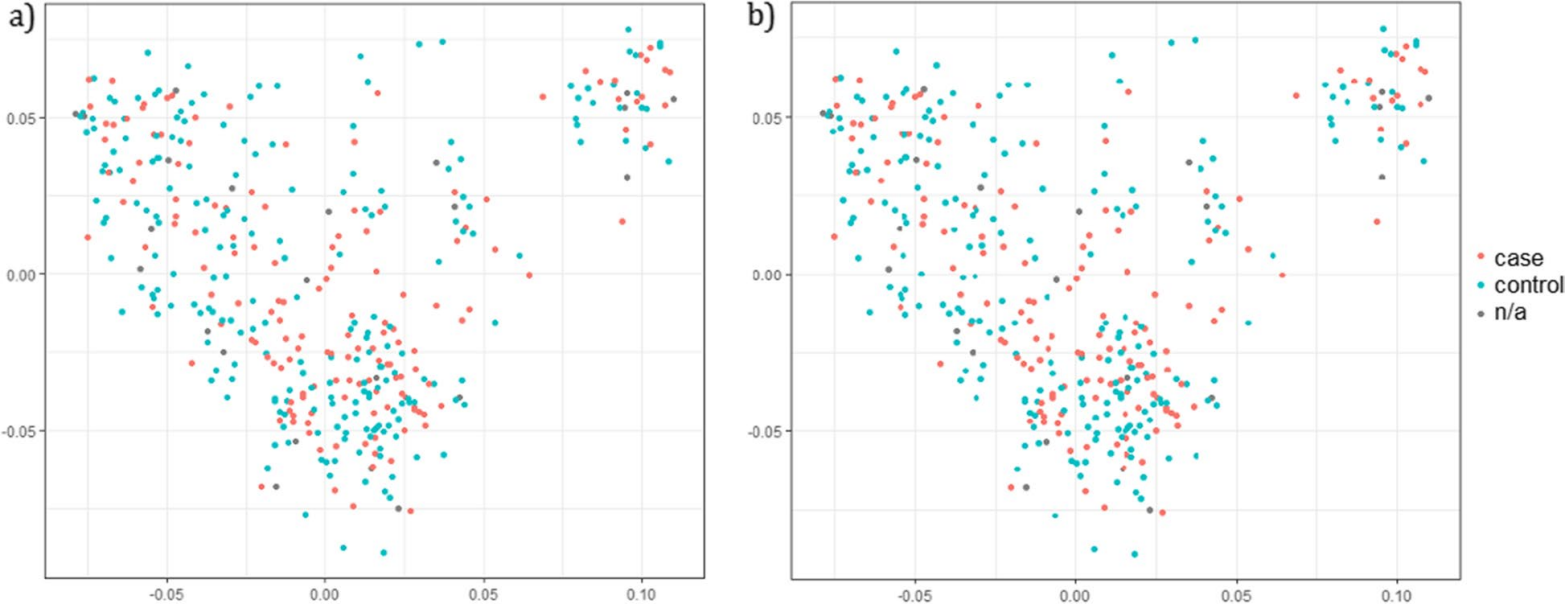
Multidimensional scaling plot for **a** firework fear and **b** noise reactivity. NA = dogs with score 2 or 3

The Multidimensional scaling (MDS)-plot show an even spread of cases and controls for both firework fear and noise reactivity (Fig. 3) and for male and female between the clusters (supplementary Fig. 1). Clustering in the population can be partly explained by subpopulations with different solid colors, but cases and controls are evenly distributed in both subpopulations (supplementary Fig. 2).

## Discussion

Our estimates showed medium to low genomic heritabilities for fear of fireworks and noise reactivity. In a previous study, pedigree-based heritability for noise reactivity and fear of fireworks was estimated to be 0.09 (noise reactivity) and 0.16 (fear of fireworks), with high genetic correlation between the two traits (r_g_ > 0.95) [51]. It has been argued that GCTA may overestimate the genomic heritability [52], but both the pedigree-based and genomic heritabilities are based on partly different and relatively small materials.

The genetic heterogeneity is lower within a dog breed than in humans, and it should therefore be easier to identify genes for complex traits with smaller materials within a single dog breed compared to humans. Still, our study supports the challenge of identifying genomic regions associated with behavior traits in general regardless of species. The region on CFA17 with a suggestive association to fear of fireworks and noise reactivity is just above the suggestive threshold with much “background noise”, as visualized on the Manhattan plots (Fig. 1). It is however notable that the two traits in question were both associated with the same region, and this region contains several genes with a potential effect on the studied traits. The results are not significant according to strict Bonferroni thresholds. Studies have argued that the traditional GWAS significance levels, using the number of tested SNPs, are too conservative in canine GWAS studies due to long LD and closely linked SNPs [48, 53]. Karlsson et al., [53] suggest that *p*-values < 2 × 10^− 05^ (depending on breed) may be an alternative to correct for the number of haplotype-blocks. The top SNPs passed the significance threshold when we used less strict criteria based on average LD of 1 Mb, 2400 blocks and *p*-values < 2 × 10^− 05^. The study is likely to be underpowered, and an increase in sample size would be preferable.

One of the genes in the candidate region, CTNNA2 (Catenin alpha 2) (Fig. 2), has been thought to be involved in bipolar disorder, a disease with a component of anxiety. The CTNNA2 gene encodes a catenin protein which is associated with several human psychiatric disorders with components of anxiety, including bipolar disorder [54] and schizophrenia [55]. The CTNNA2 gene is proposed as a candidate gene by Tang et al. (2014) in canine compulsive disorder in Doberman pinchers, a condition closely linked to anxiety [38, 56].

LRRTM1 (Leucine-rich repeat transmembrane neuronal 1) is a small protein coding gene with only 1568 base pairs. LRRTM1 is a nested gene within the bounds of the CTNNA2 gene. The LRRTM1 gene has previously been related to claustrophobia-like behavior in LRRTM1-deficient mice [57], and schizophrenia in humans [58–60].

Classification of noise reactive dogs is challenging, as a number of environmental factors influence the development and expression of the traits. The owners’ abilities to perform an “objective” and correct classification will vary, which may be a challenge as each dog has a different owner [61, 62]. Fear of fireworks might be present from early puppyhood but could also be a result of previous experiences in the dog’s life, like reactions to the smell of smoke, flashing lights, and other environmental factors. It has also been suggested that some noise-reactive dogs are suffering from physical pain in the ears or changes in auditory response [3, 63]. In general, however, owner-based questionnaires are considered an acceptable method for collecting behavioral phenotypes [64–67].

In a previous study [68], we found a correlation between the fireworks/loud noise scores and the number of fear-related behaviors observed by the owners. Dogs with scores 1 showed no fear-related behaviors, while dogs with scores 4 and 5 showed on average > 3 fear-related behaviors. None of the dogs with a score of 4 or 5 showed less than one fear-related behavior. The same study found that owners were consistent in their scoring of the same dog over time and that dogs with an initial score of 1 (controls) very rarely are reclassified to a score > 1.

Older poodles have been found to show stronger or more signs of fear than younger dogs [68] and we, therefore, had special attention to age in cases and controls. Approximately 40% of the fearful standard poodles showed signs of fear before they were 1 year old, the other 60% showed such signs, on average, before 3.9 years of age, with the median age of onset at 3. For this study, data and DNA-samples, were primarily collected from older dogs. This was done to obtain a relatively equal average age in cases and controls and to minimize the risk of false negatives in the control group. The average age of controls was 6.8 (fireworks) and 7.2 (noise), and the average age of cases was 8.2 for both traits. Thirteen dogs younger than 3 years, with a well characterized control-phenotypes, were included in the control group but removing these 13 controls did not affect the result, neither did including age as a covariate.

**Fig. 4:**
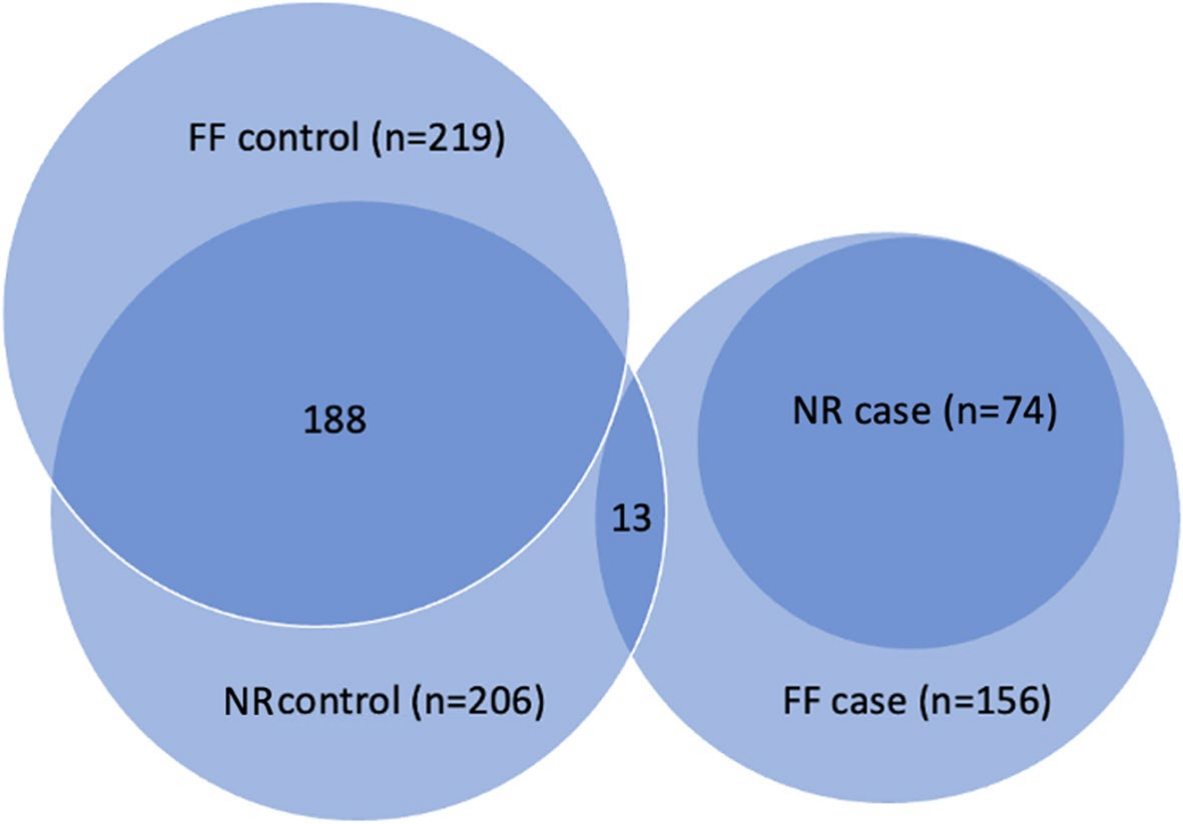
Distribution of cases (score 4 + 5) and controls (score 1) for noise reactivity (NR) and firework fear (FF) in the 400 standard poodles included in the GWAS. Dogs with scores 2 or 3 are not included

With our material, it is difficult to differentiate if fear of fireworks and noise reactivity are two distinct biological traits, but the two traits are shown to have a high correlation in standard poodles [68]. The two traits may, at least partly, be two different traits with some common risk loci. All the dogs that are described as very or extremely fearful of loud noises (cases) are also found to be very or extremely fearful of fireworks. Only about 50% of all dogs included as cases (score 4 or 5) for the fear of fireworks trait are also classified as cases in the noise reactivity trait. The majority of the dogs that are included as firework fear cases, and not included as noise reactivity cases, have a score of 2 or 3 on noise reactivity, but 13 dogs are included as firework case (score 4 or 5) and noise controls (score 1) (Fig. 4). It, therefore, seems that many of the firework cases could be reacting to the noise regardless of its origin (noise reactive), while some react exclusively or more severely to fireworks (fearful of fireworks). This could be explained if some of the reactivity to firework is caused by other stimuli, like flashing lights or the smell of gunpowder. If so, this suggest that fear of noise and fear of fireworks are, at least to some extent, two different traits, which are very hard to distinguish. That would also make it likely that many dogs are affected by both traits, reacting both to the loud noises and to other factors involved with firework fear, which complicates the phenotyping further. To distinguish between the two traits an appropriate material of dogs with fear of firework but not for noise reactivity would be needed. Unfortunately, only 13 firework cases in our material were without noise reactivity (score = 1). This was insufficient to distinguish the two traits in the analysis. We exploratively also analyzed the 82 firework cases that did not reach the inclusion criterium as noise reactivity cases, against the 219 firework controls, but in this analysis, the signal on CFA17 disappeared.

Phenotypes were collected from owner-based questionaries, where the dogs were scored from 1 (not fearful) to 5 (very fearful). To increase the likelihood of identifying associated loci, we maintained a maximum contrast between observed phenotype in cases and controls. Accordingly, dogs with a score of 2 or 3 were not included in the study (*n* = 111). Several recent studies [2, 42, 43] have included all dogs with scores > 1 as cases. One advantage of such classification is a significant increase in the number of dogs analyzed, but the disadvantage is a smaller contrast between cases and controls where owners’ ability to correctly classify the dogs’ behavior may be a challenge. By including all dogs with scores > 1 it is possible that the statistical power could be increased somewhat.

One study by Zapata [44] found an associated region of non-social fear on CFA18 in a study on 11 different breeds. Another study by Sarviaho (2019) [42],found an association of noise reactivity in an area on chromosome 20 in German shepherd dogs. Our study does not replicate those results in standard poodles. This could be due to different causal variants between the two dog breeds but also differences in the allele frequency and LD structure may affect the results.

## Conclusion

Using material from standard poodles and owner-based records, we have estimated medium and low genomic heritabilities for fear of fireworks and noise reactivity, respectively. The significant medium genomic heritability for fear of fireworks may provide helpful information for genomic selection of breeding animals in future breeding programs, which may in turn reduce the prevalence of these behavior traits. We have also identified an interesting region on CFA17, which harbors genes that have been shown to be involved in several psychiatric traits with anxiety components in humans. As in human studies, it has been challenging to identify strongly associated loci, and further research is needed to validate the suggested associated region.

## Materials and methods

### Questionnaires

Data on behavior traits in Norwegian standard poodles were collected from web- and telephone-based questionnaires. The surveys were performed in collaboration with the Norwegian poodle club (NPC) and the Norwegian Kennel Club (NKC).

The first survey (2014) had a set of questions regarding behavior and fearfulness, including fear of fireworks and noise reactivity. The second and third surveys (2017) were performed as an initial telephone survey followed by an online survey repeating the questions about fear of fireworks and noise reactivity. Finally, a fourth and fifth online questionnaire (2020 and 2022) with the same questions about fear of fireworks and loud noises was sent to owners that had not participated in the earlier studies. In all five surveys, the dog owners were asked to indicate 1) their dogs’ fear of fireworks and 2) their dogs’ reactivity to loud noises, including thunder and gunshots, on a 1 to 5 Likert scale: “Does your dog show signs of fear when exposed to firework noises?” 1 = No signs, 2 = Some signs, 3 = Obvious signs, 4 = Strong signs, 5 = Very strong signs. A translation of the relevant questions (from Norwegian) is included as supplementary Table 1.

### Included dogs

Dogs with a score of 1 (no fear) were included as controls, and dogs with a score of 4 or 5 (fearful, very fearful) were included as cases. The mean age of all dogs was 7.4 years. The mean age of controls was 6.8 (fireworks) and 7.2 (noise), with 6 being the median. Thirteen of the included controls were between 2 and 3 years old. The mean age of the cases was 8.2 for both traits, with a median of 7. The youngest included case was 1 year old. Some dogs had scores from more than one study. If the dog had more than one observation, and those scores deviated, the score from the newest online questionnaire was selected. An overview of the distribution of the selected cases and controls is given in Fig. 4.

DNA samples were collected using DNA Genotec™ Performagene PG-100 buccal swabs or EDTA blood collected by a veterinarian. All materials were gathered in accordance with the Norwegian National Committee for Research Ethics in Science and Technology’s (NENT) guidelines for research ethics in science and technology (2007). Extraction of DNA was done in accordance with Performagene 0.5 mL purification protocol using the PG-100 kit (buccal swabs) or Omega Bio-tek - E.Z.N.A® Blood DNA Mini Kit (blood). A total of 400 dogs were genotyped with the 230 K Illumina HD Canine SNP-Array (Neogen Genomics, Lincoln, NE, USA).

### Quality control

A genotyping quality control (QC) was performed. Markers with a minor allele frequency threshold (MAF) less than 0.05 and a call rate < 95% were excluded from the analyses, as well as markers failing the Hardy- Weinberg equilibrium exact test with a level of < 10^− 6^ in controls and < 10 ^− 10^ in the cases. Samples with a genotyping rate below 95% and a heterozygosity rate above three standard deviations from the mean were removed. In addition, a control for duplicates and a gender check to identify potential sample mix-ups were performed. After quality control, 145,725 SNPs remained for the noise reactivity dataset and 145,723 SNPs in the firework datasets. Three hundred eighty-five dogs remained in both datasets, including 150 cases and 212 controls for the firework phenotype and 72 cases and 200 controls for the noise reactivity phenotype (categorical trait).

### Heritability estimates

The SNP-based heritability was calculated in GCTA. A genetic relationship matrix (GRM) was calculated based on all the autosomal SNPs in the dataset. The GRM was included in a bivariate genomic restricted maximum likelihood (GREML) analysis to estimate the variance explained by the autosomal markers in the dataset using the model,

y = μ + g + e, where y is a vector of one of the two phenotypes (fear of fireworks and noise reactivity), μ is the mean term (fixed effect), g is the random genetic effect, and e is the residual error [69]. The model was tested without the youngest controls (*n* = 13).

### Genome-wide association analyses

The association analyses were performed using a mixed linear model in GCTA [69], including 145,097 autosomal markers where noise reactivity and fear of fireworks were used as dependent categorical variables, and a relationship matrix was included as a random effect to correct for relationship and population structure. Initial model testing showed limited effects of potential covariates like age, sex, coat color, and questionnaire. Manhattan plots and QQ-plots were created using R with the QQ-man package [70].

## Supplementary Information

**Additional file 1: Supplementary Fig. 1**. MDS-plot showing spread of male/female dogs. **Supplementary Fig. 2.** MDS-plot which show that the population is split in clusters that can be explained by selective breeding on solid colors where silver, brown and fawn (red) dogs tend to be bred separately from the black and white dogs.

**Additional file 2.**

## Data Availability

The datasets used and/or analyzed during the current study are available from the corresponding author on reasonable request.
