# Supplementary material for: Genomic analysis of firework fear and noise reactivity in standard poodles
Source: Canine Med Genet. 2023 Mar 8;10:2. doi: 10.1186/s40575-023-00125-0 (PMC9996964; doi:10.1186/s40575-023-00125-0)
Supplement: Supplementary file 1 — Additional file 1: Supplementary Fig. 1. MDS-plot showing spread of male/female dogs. Supplementary Fig. 2. MDS-plot which show that the population is split in clusters that can be explained by selective breeding on solid colors where silver, brown and fawn (red) dogs tend to be bred separately from the black and white dogs. [file 40575_2023_125_MOESM1_ESM.docx]

Supplementary


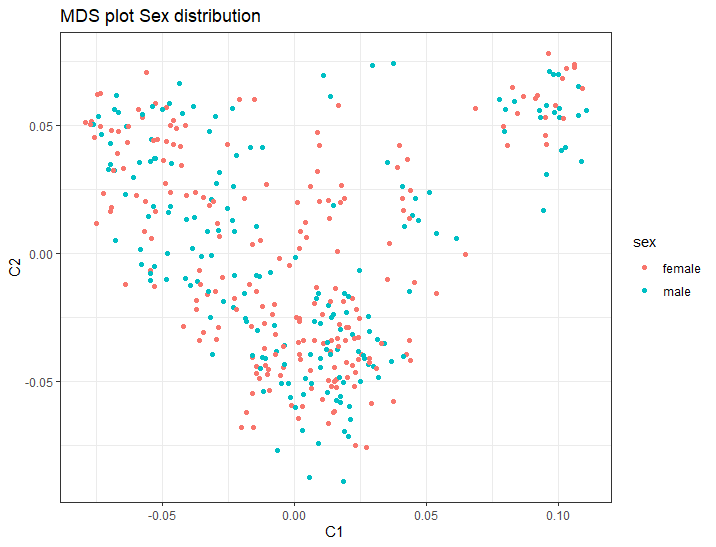


Supplementary figure 1: MDS-plot showing spread of male/female dogs.


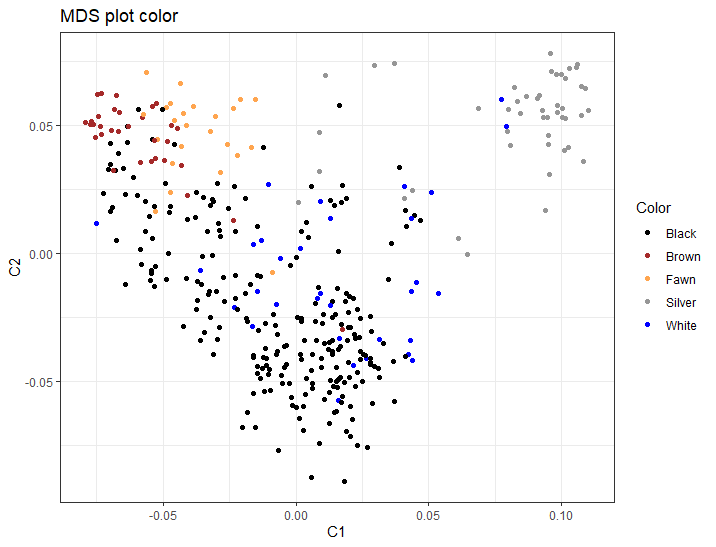


Supplementary figure 2: MDS-plot which show that the population is split in clusters that can be explained by selective breeding on solid colors where silver, brown and fawn (red) dogs tend to be bred separately from the black and white dogs.
