# Supplementary material for: Genomic analysis of firework fear and noise reactivity in standard poodles
Source: Canine Med Genet. 2023 Mar 8;10:2. doi: 10.1186/s40575-023-00125-0 (PMC9996964; doi:10.1186/s40575-023-00125-0)
Supplement: Supplementary file 2 — Additional file 2. [file 40575_2023_125_MOESM2_ESM.docx]

**Supplementary**

**The following describe the major information and questions in the questionnaires;**

**General information:**

Name of the dog, registration number, sex, date of birth, age (in years), owner name

**Is the dog in question still alive?**

Yes/No

*(Owners who replied “no” did not get questions related to DNA-sampling)*

**Does your dog show signs of fear when exposed to firework noises?**

1. No signs
2. Some signs
3. Obvious signs
4. Strong signs
5. Very strong signs
6. Don’t know

**Which of the following behaviors does your dog display when exposed to fireworks?** (Multiple selections possible)

| Shivering/trembling |
| --- |
| Pacing |
| Hiding |
| Refuse food/water |
| Fussy / clingy with owner |
| Refuse to go outdoors |
| Vocalizing |
| Salivation |
| Urinating indoors |
| Defecating indoors |
| Destroys objects  Others (please specify): |

**How long before midnight does the dog show signs of fear?**
1) More than 12 hours before
2) A few hours before
3) From the first fireworks
4) Unsure

**How long after midnight does the dog show signs of fear?**
1) Nothing after the last fireworks
2) A few hours
3) The whole night
4) Unsure

**Does your dog show signs of fear when exposed to other loud noises (gun shot, thunder, loud traffic e.g.)?**

1. No signs
2. Some signs
3. Obvious signs
4. Strong signs
5. Very strong signs
6. Don’t know

**Which of the following behaviors does your dog display when exposed to loud noises?** (Multiple selections possible)

| Shivering/trembling |
| --- |
| Pacing |
| Hiding |
| Refuse food/water |
| Fussy / clingy with owner |
| Refuse to go outdoors |
| Vocalizing |
| Salivation |
| Urinating indoors |
| Defecating indoors |
| Destroys objects  Others (please specify): |
